# Effect of the Addition of the Fifth Amino Acid to [GADV]-Protein on the Three-Dimensional Structure

**DOI:** 10.3390/life13010246

**Published:** 2023-01-16

**Authors:** Koichi Kato, Tomoki Nakayoshi, Ryota Oyaizu, Natsuko Noda, Eiji Kurimoto, Akifumi Oda

**Affiliations:** 1 Faculty of Pharmacy, Meijo University, 150 Yagotoyama, Tempaku-ku, Nagoya 468-8503, Japan; 2 Faculty of Pharmaceutical Sciences, Shonan University of Medical Sciences, 16-48 Kamishinano, Totsuka-ku, Yokohama 244-0806, Japan; 3 Faculty of Pharmacy, Kinjo Gakuin University, 2-1723 Omori, Moriyama-ku, Nagoya 463-8521, Japan; 4 Graduate School of Information Sciences, Hiroshima City University, 3-4-1 Ozukahigasi, Asaminami-ku, Hiroshima 731-3194, Japan; 5 Institute for Protein Research, Osaka University, 3-2 Yamadaoka, Suita 565-0871, Japan

**Keywords:** primitive protein, molecular dynamics simulation, [GADV]-peptide, structural prediction

## Abstract

The [GADV]-protein, consisting only of glycine (G), alanine (A), aspartic acid (D), and valine (V), is frequently studied as a candidate for a primitive protein that existed at the beginning of life on Earth. The number of proteogenic amino acids increased during evolution, and glutamic acid may have been added as the fifth amino acid. In this study, we used molecular dynamics simulations to estimate the conformation of random peptides when glutamate is added to G, A, D, and V ([GADVE]), when leucine is added ([GADVL]), and when the frequency of alanine is doubled ([GADVA]). The results showed that the secondary structure contents of the [GADVE]-peptide and [GADVL]-peptide were higher than that of the [GADVA]-peptide. Although the [GADVL]-peptide had a higher secondary structure formation ability than the [GADVE]-peptide, it was less water soluble, suggesting that it may not be a primitive protein. The [GA(D/E)V]-peptide with G:A:D:V:E = 2:2:1:2:1 according to the occurrence ratio in the codon table also increased the secondary structure contents compared to the [GADV]-peptide, indicating that the addition of glutamic acid increased the structure formation ability of the primitive protein candidates.

## 1. Introduction

Several hypotheses have been proposed about the molecules used to create the first life on Earth at the Hadean eon. These hypotheses, including the RNA world hypothesis [1], the protein world hypothesis [2,3,4], and the garbage bag world hypothesis [5], have strengths and weaknesses, and no conclusive evidence has been obtained. However, all the hypotheses suggest that biomolecules such as proteins and nucleic acids were included in the early stages of life. Since all living organisms on Earth today are composed of proteins and nucleic acids, these molecules must have been used to construct organisms at least as early as the last universal common ancestor (LUCA) [6,7]. In contrast, at the Hadean eon, proteins and nucleic acids were unlikely to be constructed by the complex and elaborate system, the same as today’s organisms. It is often proposed that the protein-synthesizing system and the nucleic-acid-synthesizing system evolved by influencing each other [8,9,10,11].

Primitive life is unlikely to have had the same elaborate biopolymers as today; the genetic code may have been different from today’s. Moreover, there is a hypothesis that the universal genetic code, which consists of 4^3^ = 64 types of codons with four nucleic acids, may not have been complete from the beginning of life [11]. Therefore, the constituent amino acids of primitive proteins might be different from those of today’s proteins. Proteins in living organisms are composed of about 20 types of amino acids (Magic 20), but many hypotheses proposed that not all of the Magic 20 existed at the beginning of life. In these hypotheses, primitive proteins comprised only a limited variety of amino acids [2,3,11,12,13,14,15,16]. There are two methods for estimating the components of a primitive protein: one is to examine 3D structures of peptides constructed by a limited type of amino acids [12,14], and the other is to replace residues of a currently existing protein with simpler amino acids while preserving the protein functions [15,16]. In the previous studies, the former method was used to estimate the first proteins of life, and the latter was used to estimate the protein components of LUCA.

Glycine (G), alanine (A), aspartate (D), and valine (V) are recognized as components of primitive proteins [2,3,17,18]. All have relatively simple structures and are easy to synthesize from inorganic materials. Moreover, they were found in space-derived amino acids [19,20,21] and in amino acids obtained from Miller’s experiments [22] (and its modifications [23]), suggesting that they could have existed in the primitive Earth environment. Moreover, since the GADV amino acid set includes both hydrophilic and hydrophobic amino acids, and these are relatively easy to form secondary structures, the [GADV]-protein comprising only four amino acids, G, A, D, and V is often considered to be the earliest protein in primitive life. Since G, A, D, and V are encoded by the genetic code GNC (N = A, C, G, U) in current life; they may be linked to the origin of the genetic code [2,3]. Thus, the importance of the [GADV]-protein is considered regardless of which theory is adopted as the origin of life [17,18]. Meanwhile, glutamic acid (E) is proposed as the “fifth” amino acid of primitive proteins [11] because E is the amino acid that appears when the third cytosine of codon GNC is replaced by adenine or guanine (Table 1).

We have previously performed molecular dynamics (MD) simulations to clarify the conformation of proteins using only a limited number of amino acids [24,25,26]. MD simulations of [GADV]-peptides, in which G, A, D, and V are randomly arranged, can form a protein secondary structure [24,25]. In the evaluation, we calculated the secondary structure formation, structural fluctuations of the peptide, polar surface area, etc., to estimate the “protein-like” properties of the peptide, i.e., easiness to inherent structure formation. Whichever hypothesis of the origin of life is adopted (RNA world, protein world, or garbage bag world hypotheses, etc.), it is sometimes proposed that the system of replication was not perfectly implemented at the beginning of life and that randomly formed peptides played an important role [4,17,18,27,28]. Therefore, such random peptides may have been the ancestors of proteins. We also estimated the 3D structure of peptides containing d-amino acids and l-amino acids. We found that it is easier to obtain a protein-like structure in an environment where only one of the optical isomers is concentrated than in an environment where only racemic amino acids are included [25]. These studies investigated the components of the primitive protein from the viewpoint of the three-dimensional structure of primitive proteins. This study used the same approach to presume the “fifth” amino acid, after G, A, D, and V, in the primitive protein.

## 2. Materials and Methods

This study performed MD simulations to estimate the 3D structure of random peptides consisting of 20 residues. Random peptides of the [GADVE] and [GADVL] amino acid sets were constructed by adding E or leucine (L), respectively, to the [GADV] set. Although L is unlikely to have existed on primitive Earth, it is easily included in secondary structures [29]. Therefore, we used [GADVL] to compare the secondary structure-forming ability of other sets. Furthermore, during evolution, the fifth amino acid must make it easier to create a protein 3D structure than the [GADV] set. Thus, we performed MD simulations for the [GADV] set for comparison. For these, the frequency of appearance of each amino acid residue was assumed to be the same. Therefore, the appearance rates of amino acids were 1:1:1:1 for the [GADV] set and 1:1:1:1:1 for the [GADVE] and [GADVL] amino acid sets. A total of 100 random peptides were made for each set, and their ability to form 3D structures was evaluated.

When comparing the [GADV] set with the [GADVX] set, it is unclear whether the difference is due to amino acid X or to the reduced ratio of G, which is less likely to form secondary structures. Therefore, we also performed MD simulations for the [GADVA] set (G:A:D:V = 1:2:1:1), in which the frequency of A was doubled. Additionally, if nucleic acids were created before proteins and a primitive translation mechanism using codons existed, the frequency of appearance of D and E may have been half that of G, A, and V (Table 1). Therefore, we also evaluated the [GA(D/E)V] set, where the frequency is G:A:D:V:E = 2:2:1:2:1.

For MD simulations, we used AMBER16 [30] and AMBER ff12SB force field. We calculated under continuum solvent using the generalized Born method GBneck2 (igb = 8) [31]. For the atomic radius, we used mbondi3, a time-step of 1 fs, and a total simulation time of 200 ns at a temperature of 300 K. The structured output was available every 0.5 ps. SHAKE was used for the bonds, including hydrogen atoms. All carboxyl groups of D, E, and C-termini were negatively charged. The amino groups of N-termini were positively charged.

In the analysis of the results, the last 10 ns (20,000 fames) of the MD trajectory was used. First, the cpptraj module of AmberTools was used to analyze secondary structure formation, structural fluctuations, and hydrogen bond formation. For secondary structure formation analyses, the program DSSP [32] was used, and α-helices, π-helices, and 3–10 helices were regarded as “helix structures”. In contrast, most of the β-structures were turn structures, and there were no stably retained sheet structures because peptides include only 20 residues. Root-mean-square fluctuation (RMSF) was calculated using the average structure of the last 10 ns trajectories as a reference for structural fluctuation analyses. For hydrogen bonding analyses, hydrogen bonds were defined as pairs of atoms with X-H…X’ angles of 120° or more and distances X…H of 3.5 Å or less. In addition to the analyses by cpptraj, the solvent accessible surface area (SASA) was calculated by the DMS [33,34] program. In addition to the total surface area of the molecule, the polar surface area (PSA) was also calculated, and the trajectory average was obtained. The PSA was used as an indicator of hydrophilicity [35].

## 3. Results and Discussion

**Figure 1:**
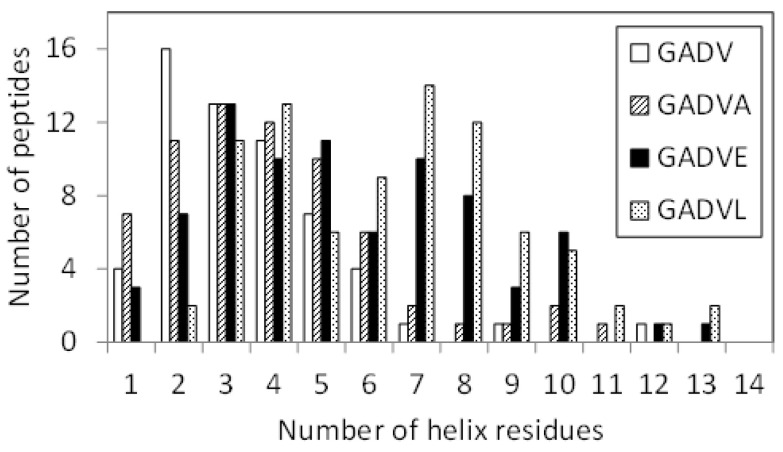
Classification of peptides according to numbers of helix residues. Histogram showing numbers of the peptides with *n* helix residues.

Since E is proposed as the fifth amino acid of primitive proteins [11], we examined the structure formation ability of [GADVE]-peptides and compared its ability with those of [GADV]-, [GADVA]-, and [GADVL]-peptides. The secondary structure formation abilities of [GADV]-, [GADVA]-, [GADVE]-, and [GADVL]-peptides were compared in which the residue is considered to form the secondary structure if any residue maintained a particular secondary structure in 50% of the frames in the last 10 ns trajectory of the MD simulation. The number of such secondary structure-forming residues in each peptide was counted. Since no residues formed the β-sheet structure in 50% of the 10 ns trajectories described above, the helix-forming residues are shown in Figure 1, where a histogram was created by classifying 100 peptides by the number of helix residues. Although this figure illustrated the number of peptides including at least one helix residue, the number of peptides with no helix residues was 42 for the [GADV]-peptide, 34 for the [GADVA]-peptide, 21 for the [GADVE]-peptide, and 17 for the [GADVL]-peptide. The average number of helix-forming residues was 2.06 for the [GADV]-peptide, 2.64 for the [GADVA]-peptide, 4.33 for the [GADVE]-peptide, and 5.31 for the [GADVL]-peptide. Therefore, the [GADVA]-peptide has a higher helix formation capacity than the [GADV]-peptide reflecting that the lower G content is less likely to form secondary structures. In contrast, when [GADVA]-, [GADVE]-, and [GADVL]-peptides with the same G content are compared, more helix is formed when E and L are introduced instead of A. This indicates that adding the “fifth” residue facilitates the formation of protein-like secondary structures such as helices, which may have been the driving force behind the increase in the variety of protein-forming amino acids during evolution. Furthermore, the [GADVL]-peptide is more likely to form a helix than the [GADVE]-peptide, reflecting the difference in the ability of E and L to form secondary structures.

**Figure 2:**
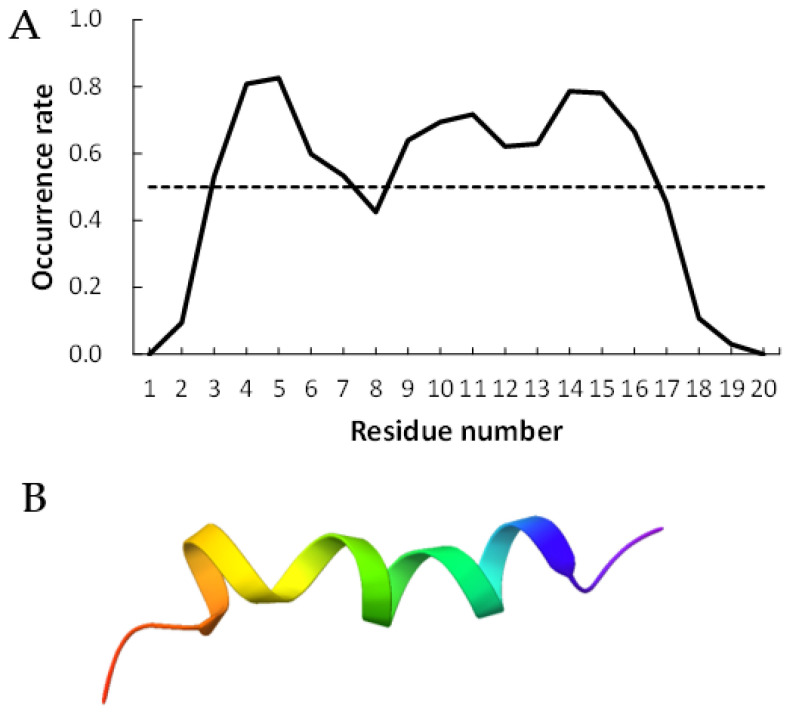
Example of helix-forming [GADVE]-peptides. The sequence of the peptide is GDEVVAEGEVAAADEEEGAG. (**A**) The occurrence rate of helix formation for the last 10 ns of simulations. The dotted line shows a 50% occurrence. (**B**) Structure of the example of [GADVE]-peptide.

**Figure 3:**
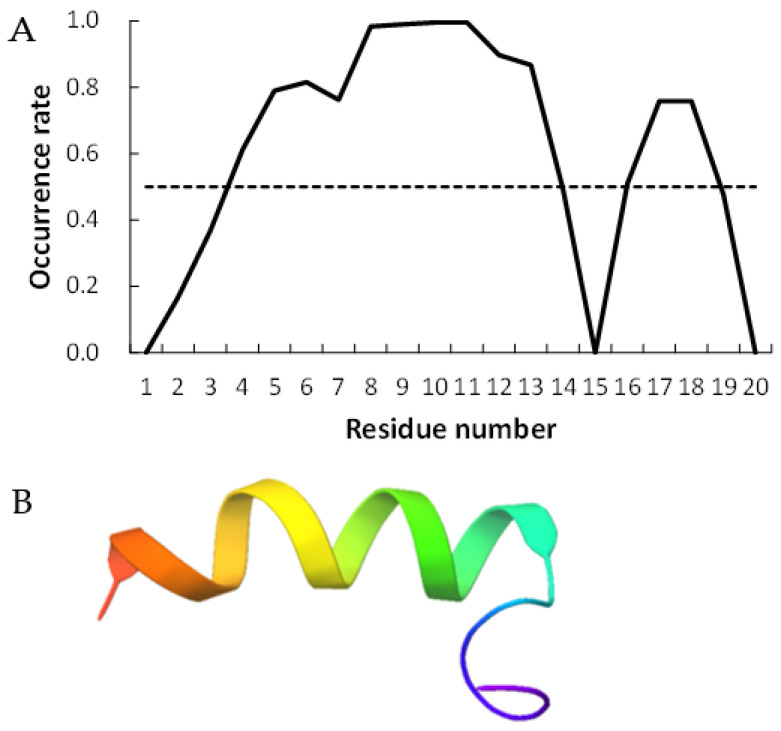
Example of helix-forming [GADVL]-peptides. The sequence of the peptide is GDAVLDLLLVLLLLALGAVVLV. (**A**) The occurrence rate of helix formation for the last 10 ns of simulations. The dotted line shows a 50% occurrence. (**B**) Structure of the example of [GADVL]-peptide.

Figure 2 and Figure 3 show examples of peptides with the most helix-forming residues in the [GADVE]-peptide and the [GADVL]-peptide, respectively. The former sequence was GDEVVAEGEVAAADEEEGAG with 13 helix residues during 50% of the last 10 ns of the simulation. The latter sequence was GDAVLDLLLVLLLLALGAVVLV, which also had 13 helix residues. The occurrence frequency of the helix structure at each residue is also shown. Therefore, even randomly generated peptides can construct helix structures of some length. Similarly, the [GADV]- and [GADVA]-peptides also had peptides that formed a helix of some length, consistent with the hypothesis that the [GADV]-peptides were the earliest primordial proteins. However, adding the “fifth” amino acid increases the secondary structure formation ability, and it may be one of the causes of an increase in the variety of proteogenic amino acids. In other words, when considering the “primordial soup” of amino acids, a soup containing five amino acids is more likely to form proteins than a soup containing only four amino acids. It suggests that the secondary structure formation ability may be a selection pressure that led to the evolution from a four-amino-acid soup to a five-amino-acid soup.

**Figure 4:**
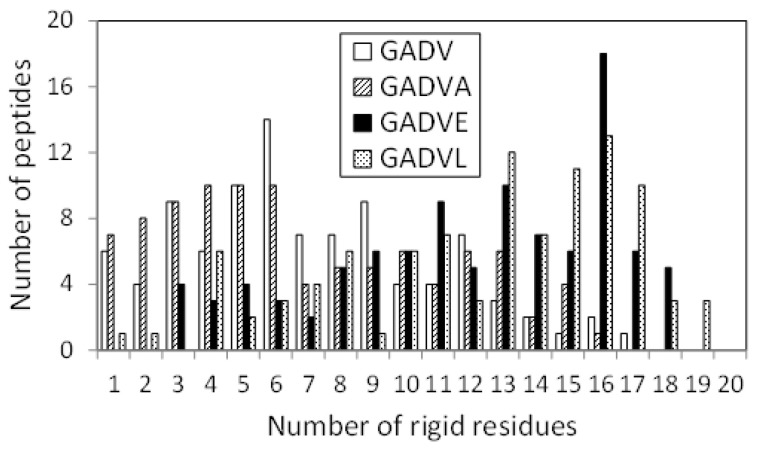
Histogram showing the numbers of peptides with *n* rigid residues.

The 3D structure of the peptides with limited amino acids has been evaluated for their secondary structure formations. However, there is no evidence that primitive proteins using only a limited number of amino acids formed a secondary structure similar to that of now-existing proteins, and they may have some unique ordered structure. Since the program DSSP evaluates the secondary structure of existing proteins, the ordered structures, which are different from the general secondary structures, must be assessed by another method. Therefore, we calculated the RMSF for each atom of the peptide and evaluated its fluctuation. If a peptide has some ordered structure, its atoms are less likely to fluctuate. In this study, residues with RMSF of Cα less than 4.0 Å were defined as “rigid residues”, and the number of such residues evaluated the rigidity of peptides. Figure 4 shows the histograms of the [GADV]-, [GADVA]-, [GADVE]-, and [GADVL]-peptides classifying according to the number of rigid residues. Same as in Figure 1, only peptides with at least one rigid residue were counted, but there were four, three, one, and one peptide with no rigid residues for the [GADV]-, [GADVA]-, [GADVE]-, and [GADVL]-peptides, respectively. Moreover, the average number of rigid residues was 6.84 for the [GADV]-peptide, 6.71 for the [GADVA]-peptide, 11.87 for the [GADVE]-peptide, and 12.21 for the [GADVL]-peptide. The average numbers of the rigid residues were similar between the [GADV]- and [GADVA]-peptides, and the average values of the Cα RMSFs were also close (4.45 Å for the [GADV]-peptide and 4.48 Å for the [GADVA]-peptide). The [GADVA]-peptide with a lower occurrence ratio of G is more likely to form a secondary structure, but the [GADV]-peptide is comparable to the [GADVA]-peptide for peptide rigidities (Figure 1). The result indicates that the [GADV]-peptide can form rigid conformations as primitive proteins. The [GADVE]- and [GADVL]-peptides had more rigid residues than the [GADV]- and [GADVA]-peptides, and the average RMSF of Cα was 4.05 Å for the [GADVE]-peptide and 3.87 Å for the [GADVL]-peptide, indicating rigid overall structure. Thus, even if not only the secondary structure but also other ordered structures are considered, the addition of the “fifth” amino acid is advantageous in the structure formation of the primordial protein. Furthermore, the comparison between [GADVE]- and [GADVL]-peptides suggests that [GADVL]-peptides form a more rigid structure. It may reflect the hydrophobicity of L. In the [GADVL]-peptide, there were three examples in which 19 of the 20 residues were rigid, indicating that high hydrophobicity is highly advantageous in forming ordered structures.

The average number of hydrogen bonds formed per peptide was calculated in the 100 peptides created for each set of amino acids. The results were 7.21, 7.07, 8.13, and 8.73 hydrogen bonds for the [GADV]-, [GADVA]-, [GADVE]-, and [GADVL]-peptides, respectively. This corresponds to each amino acid set’s secondary structure formation ability and rigidity, indicating that adding the “fifth” amino acid promotes the formation of rigid 3D structures, including hydrogen bond formation.

Therefore, the [GADVL]-peptides are most favorable in forming protein-like conformations. However, it is usually assumed that primitive life was generated in the water solvent because hydrocarbons and other organic solvents were unavailable in large quantities on primitive Earth. Thus, it is necessary to consider whether the [GADVL]-peptide could have properties appropriate for primitive life on the primitive water environment of the Earth.

**Figure 5:**
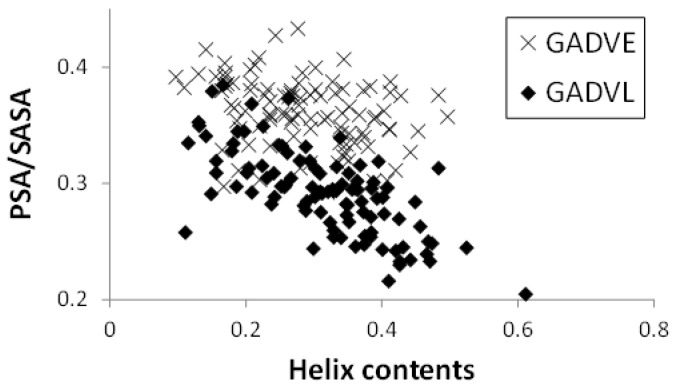
Relationship between PSA/SASA and helix contents of peptides.

Figure 5 shows the relationship between the helix content and the PSA/SASA ratio of the peptides in the [GADVE]- and [GADVL]-peptides. In this figure, one point represents one peptide, and 100 points are plotted for each set of amino acids. The helix content was higher for the [GADVL]-peptide (0.286 for the [GADVE]-peptide and 0.316 for the [GADVL]-peptide on average), but not so large. In contrast, the [GADVL]-peptide showed a stronger correlation between helix content and PSA/SASA than the [GADVE]-peptide, with a correlation coefficient of −0.313 for the [GADVE]-peptide versus −0.698 for the [GADVL]-peptide. This means that helix-forming peptides are less water-soluble, and the [GADVL] amino acid set is less appropriate for forming water-soluble peptides with a rigid structure.

Furthermore, the high correlation coefficient suggests that even if water-soluble [GADVL]-peptides were produced, they would likely form similar structures, which may not be desirable in the diversity of biomolecules. L is an amino acid that is rarely thought to have existed in the primitive terrestrial environment, and it was suggested that it may not be appropriate as an amino acid constituting the earliest proteins in terms of the balance between water solubility and 3D structure formation. The correlation coefficient was −0.350 for the [GADV]-peptide and −0.507 for the [GADVA]-peptide, both lower than that of the [GADVL]-peptide. This indicates that a certain degree of structural diversity can be achieved by the [GADV] amino acid set. The [GADVE]-peptide has a similar correlation coefficient to the [GADV]-peptide, suggesting that the [GADVE]-peptide has a high ability to form 3D structures without decreasing the possibility of forming water-soluble peptides with various 3D structures. Thus, adding E as a “fifth” amino acid may promote the formation of the primitive protein.

**Figure 6:**
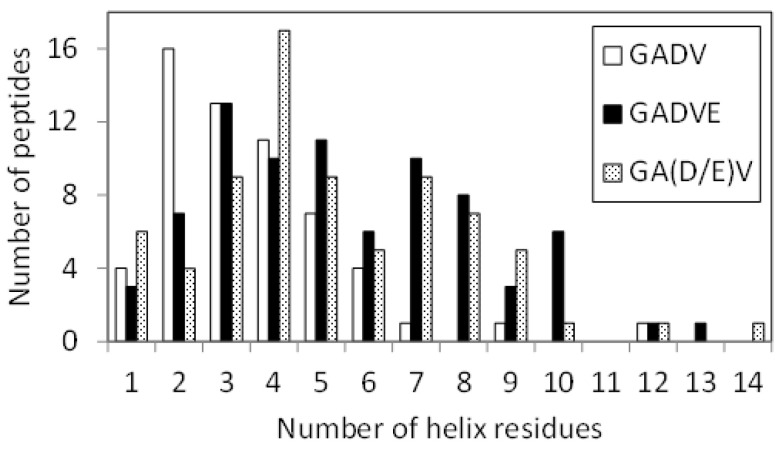
Histogram showing the number of peptides with *n* helix residues.

If we consider the set of amino acids consisting of G, A, D, V, and E as the building blocks of a primitive protein, and if codons were already present at the time of formation of the [GADVE]-protein, the ratio of G, A, and V to D and E could have been twice as high as that of D and E (see Table 1). Therefore, we also investigated the conformation of the [GA(D/E)V]-peptide, in which the ratio of D and E is half of G, A, and V. If the [GA(D/E)V]-peptides are unable to form a protein-like conformation, it is possible that the translation system did not exist at the time E was added as the “fifth” amino acid. Figure 6 shows the helix-forming ability of the [GADV]-, [GADVE]-, and [GA(D/E)V]-peptides. This is a similar histogram to Figure 1. The number of peptides with no helix residues was 42, 21, and 26 for the [GADV]-, [GADVE]-, and [GA(D/E)V]-peptides, respectively. The average number of helix residues was 2.06, 4.33, and 3.84 for the [GADV]-, [GADVE]-, and [GA(D/E)V]-peptides, respectively. The [GA(D/E)V]-peptide, although inferior to the [GADVE]-peptide, has a higher secondary structure-forming ability than the [GADV]-peptide, and the addition of E as the “fifth” amino acid gives it a protein-like structure. Unlike the [GADVE]-peptide, the ratio of G, A, and V were not changed in the [GA(D/E)V]-peptide in comparison with the [GADV]-peptides. Nevertheless, the secondary structure-forming ability is increased, suggesting the importance of E in primitive protein formation. Notably, the [GA(D/E)V]-peptide outperforms even the [GADVA]-peptide (Figure 1), which has a lower ratio of G present, indicating that adding E is more important in secondary structure formation than reducing G.

**Figure 7:**
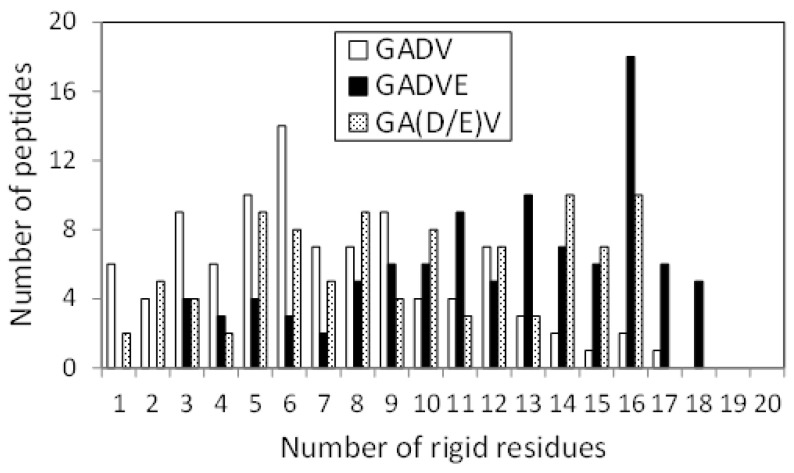
Histogram showing the number of peptides with *n* rigid residues.

Figure 7 shows the histograms of the [GADV]-, [GADVE]-, and [GA(D/E)V]-peptides classifying according to the number of rigid residues, similar to Figure 4. The numbers of peptides that have no rigid residues were four, one, and four for the [GADV]-, [GADVE]-, and [GA(D/E)V]-peptides, respectively. The average numbers of rigid residues were 6.84, 11.87, and 9.09 for the [GADV]-, [GADVE]-, and [GA(D/E)V]-peptides, respectively. In addition to the number of helix residues, the number of rigid residues of [GA(D/E)V]-peptides was larger than that of [GADV]-peptides. The result indicates that the structure-forming ability of the [GA(D/E)V]-peptides were superior to that of the [GADV]-peptides, even if not only ordinary secondary structures but also non-standard rigid structures were considered. Because the number of rigid residues of [GADVA]-peptides was similar to that of [GADV]-peptides (Figure 4), the [GA(D/E)V]-peptides can form more rigid structures than the [GADVA]-peptides, despite of the large proportion of the flexible amino acid G. The average number of hydrogen bonds in the [GA(D/E)V]-peptides was 7.61, which was more significant than that in the [GADV]-peptides. The result seems to reflect the secondary structure formation ability and/or rigid structure formation ability of the [GA(D/E)V]-peptides.

**Figure 8:**
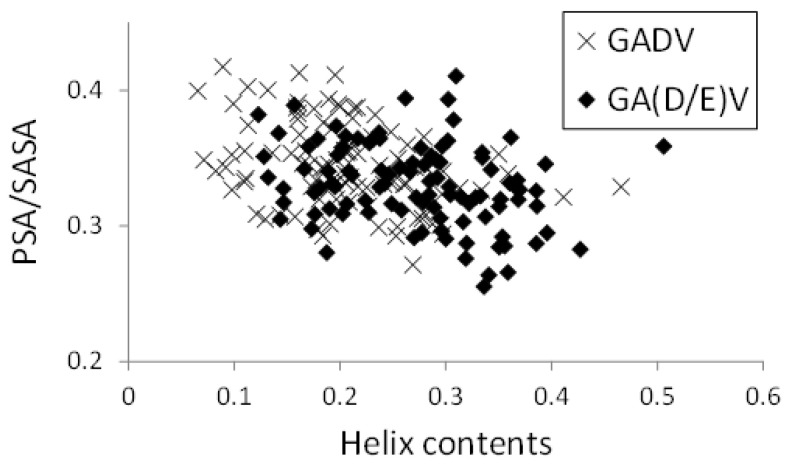
Relationship between PSA/SASA and helix contents of peptides.

In Figure 8, the relationships between helix contents and PSA/SASA for the [GADV]- and [GA(D/E)V]-peptides were illustrated, similar to Figure 5. Although the helix contents of the [GA(D/E)V]-peptides were larger than that of the [GADV]-peptides (average values were 0.272 and 0.207 for [GA(D/E)V] and [GADV], respectively), PSA/SASA was similar (0.330 for [GA(D/E)V] and 0.345 for [GADV]). Moreover, the correlation coefficients between PSA/SASA and helix contents were −0.277 and −0.351 for the [GA(D/E)V]- and [GADV]-peptides, respectively, and it indicates that the introduction of a “fifth” amino acid is advantageous for the structural variety of primitive proteins. Although the structure formation ability of the [GA(D/E)V]-peptides was less than that of the [GADVE]-peptides, the structures of the [GA(D/E)V]-peptides were more protein-like than those of the [GADV]-peptides. Therefore, the possibility that the “[GA(D/E)V]-peptide world” evolved from the “[GADV]-peptide world” cannot be ruled out because the [GA(D/E)V]-peptide is assumed to be evolutionally generated after the birth of the genetic code. These results were consistent with the protein world and nucleic acid world hypotheses, and comparing the structure formation abilities cannot clarify whether the protein world hypothesis or the RNA world hypothesis is correct. However, our findings suggest that the glutamic acid residue is vital in the primitive proteins in addition to G, A, D, and V.

## 4. Conclusions

In this study, we focused on glutamic acid as a possible “fifth” amino acid to be added to the [GADV]-peptide, which is recognized as the earliest protein. The role of E in the protein structure was estimated by MD simulation of random peptides, including G, A, D, V, and E. The results indicate that more protein-like structures can be formed by adding E to the [GADV]-peptides, although E is the hydrophilic amino acid. Because the proportion of the simplest amino acid G is reduced in [GADVX]-peptides compared with [GADV]-peptides, [GADVX]-peptides were compared with not only [GADV]-peptides but also [GADVA]-peptides, in which the proportion of G was the same as the [GADVX]-peptides. More secondary structures were formed in [GADVE]-peptides than in [GADVA]-peptides. Moreover, even in [GA(D/E)V]-peptides, in which the proportion of G was similar to [GADV]-peptides, more secondary structures and rigid residues were formed than in [GADVA]-peptides. These results suggest that adding E for the presumed primitive proteins increases the protein 3D structure formation ability. Although more rigid structures were found in [GADVL]-peptides than in [GADVE]-peptides, the correlation coefficient between helix contents and PSA/SASA was higher in [GADVL]-peptides than in [GADVE]-peptides. The result indicates that secondary structure-forming [GADVL]-peptides have low water-solubilities, and [GADVE]-peptides were preferable to [GADVL]-peptides for the presumed primitive proteins.

## Figures and Tables

**Table 1:** Amino acids corresponding to codon GNN.

Codon	Amino Acid	Codon	Amino Acid	Codon	Amino Acid	Codon	Amino Acid
GUU	Val	GCU	Ala	GAU	Asp	GGU	Gly
GUC		GCC		GAC		GGC	
GUA		GCA		GAA	Glu	GGA	
GUG		GCG		GAG		GGG	

## Data Availability

Data are contained within this article.
